# Establishment and Characterization of a Transgenic Mouse Model for In Vivo Imaging of Bmp4 Expression in the Pancreas

**DOI:** 10.1371/journal.pone.0024956

**Published:** 2011-09-15

**Authors:** Mayu Yasunaga, Nao Oumi, Mitsuhiko Osaki, Yasuhiro Kazuki, Tomoko Nakanishi, Mitsuo Oshimura, Kenzo Sato

**Affiliations:** 1 Division of Molecular Biology, School of Life Sciences, Faculty of Medicine, Tottori University, Yonago, Japan; 2 Department of Biomedical Science, Faculty of Medicine, Institute of Regenerative Medicine and Biofunction, Tottori University, Yonago, Japan; 3 Chromosome Engineering Research Center, Tottori University, Yonago, Japan; University of Bremen, Germany

## Abstract

Type-2 diabetes results from the development of insulin resistance and a concomitant impairment of insulin secretion. Bone morphogenetic protein 4 (Bmp4)-Bmp receptor 1A signaling in β cells has recently been reported to be required for insulin production and secretion. In addition, Bmp4 blocks the differentiation and promotes the expansion of endocrine progenitor cells. Bmp4 therefore regulates the maintenance of homeostasis in the pancreas. In this study, we constructed a reporter plasmid carrying 7-kb enhancer and promoter region of the Bmp4 gene upstream of the firefly luciferase gene. We used this construct to produce transgenic mice by pro-nuclear microinjection, for subsequent in vivo monitoring of Bmp4 expression. The bioluminescent signal was detected mainly in the pancreas in three independent lines of transgenic mice. Furthermore, the bioluminescent signal was enhanced in association with the autophagy response to 24-h fasting. These results suggest that pancreatic expression of Bmp4 is involved in responding to the physiological environment, including through autophagy. These mouse models represent useful tools for toxicological screening, and for investigating the mechanisms responsible for pancreatic Bmp4 functions in vivo, with relevance to improving our understanding of pancreatic diseases.

## Introduction

Healthy aging is thought to reflect the combined influences of environmental factors (e.g., lifestyle choices) and genetic factors. Human longevity correlates with the avoidance of age-related diseases that are associated with a variety of multiorgan complications, such as diabetes [1]. Diabetes develops as a consequence of insulin resistance and impaired β-cell function. A progressive loss of β-cell function and β-cell mass triggers the transition from glucose intolerance to overt diabetes during the course of the disease [2], [3]. The intracellular signaling mechanisms regulating insulin secretion have been extensively studied [4], and transcription factors have been linked to β-cell dysfunction in type 2 diabetes and maturity-onset diabetes of the young [3], [5]. In contrast, information on real-time monitoring of the expression of genes involved in glucose homeostasis in pancreatic cells is lacking. More information on the monitoring and analysis of pancreatic functions that maintain glucose homeostasis is therefore required to help prevent pancreatic diseases and promote healthy aging.

Bone morphogenetic protein 4 (Bmp4) is a multifunctional growth factor that belongs to the transforming growth factor β superfamily. Bmp4 is essential for mouse development, and most Bmp4-null mouse embryos die at the onset of gastrulation, as a result of failure of mesodermal development [6]. Bmp4 has been reported to perform two functions in the pancreas. [7]–[10]. Bmp4 and Bmp Receptor 1A (BmpR1A) are expressed in pancreatic β cells, and autocrine Bmp4 signaling induces Smad signaling through the receptor complex (at least one BmpR1A and BmpR2). Mice with attenuated BmpR1A signaling in β cells show decreased expression of key genes involved in insulin gene expression, proinsulin processing, glucose sensing, secretion-stimulus coupling, incretin signaling, and insulin exocytosis; these mice consequently develop diabetes as a result of impaired insulin secretion [8], [9]. Moreover, heterozygous knock-out BmpR1A mice demonstrate abnormal glucose metabolism [8], [9].

However, a recent report showed that Bmp4 stimulation blocked the differentiation and promoted the expansion of endocrine progenitor cells, thereby revealing a novel paradigm of signaling explaining the balance between expansion and differentiation through regulation of Id2 in pancreatic duct epithelial progenitors [10]. Likewise, Bmp4 has been reported to enhance mouse embryonic stem cell self-renewal [11], and is necessary for the production of hematopoietic progenitors [12].

Bmp4 thus exhibits divergent and complex functions by interacting with many cells and organs in vivo, including regulating the maintenance of homeostasis in the pancreas. In this study, we aimed to establish transgenic mice using pancreatic-specific regions 7 kb upstream of the transcription start site to allow the monitoring of Bmp4 expression for toxicological screening in the pancreas. In vivo Bmp4 expression was serially monitored using bioluminescence imaging, based on an optical technique generally used for the quantification of cell mass. Bioluminescence refers to the enzymatic generation of visible light by living organisms. The enzymatic catalysis of the luciferase substrate D-luciferin results in the emission of photons at wavelengths that can be detected by a photosensitive detector, making the technique suitable for imaging biological processes under a variety of conditions [13]–[15].

In this study, we established transgenic mice that allowed us to monitor Bmp4 expression by bioluminescence imaging for toxicological screening in vivo. This system enabled the long-term, quantitative, monitoring of Bmp4 expression in the pancreas, and allowed us to demonstrate changes in Bmp4 associated with fasting and autophagy.

## Results

### Luciferase expression of Bmp4Luc reporter plasmids in pancreatic cell lines

**Figure 1:**
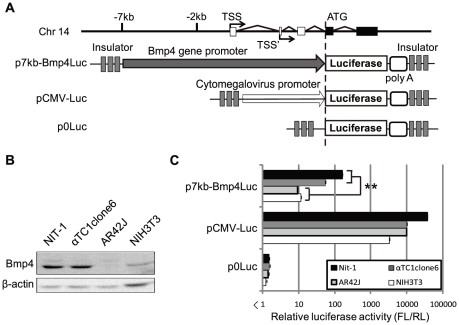
Construction of p7kb-Bmp4Luc and luciferase activities. The mouse Bmp4 is located on the long arm of mouse chromosome 14, and has two transcription start sites (TSS, designated also as 0, TSS'). (A) The reporter vector (p7kb-Bmp4Luc) contains the promoter/enhancer region (7 kb), as well as 5′noncoding exons and introns of the Bmp4 gene. The Bmp4Luc expression unit was flanked on either side by chicken globin gene insulators. The translation initiation site of the firefly luciferase gene was adjusted to that of Bmp4. pCMV-Luc was constructed using the cytomegalovirus early promoter, as a control vector. p0Luc contained no promoter or enhancer. (B) Endogenous expression of Bmp4 and β-actin in NIT-1, αTC1clone6, AR42J and NIH3T3 cells, determined by western blotting analysis. (C) Luciferase assays. Each cell was transiently cotransfected with reporter plasmids and *Renilla* luciferase vector (pRL-TK) as a transfection control. The *x* axes show relative luciferase activities: firefly luciferase (FL) from the reporter plasmid was normalized to *Renilla* (RL) luciferase activity from the control vector. All data are shown as mean ± SE from three independent experiments. Asterisk (**) represents significant difference (p<0.01) by Student's *t*-test.

Little is known about the transcriptional regulation of the *Bmp4* gene in vivo and in vitro. A 2.4-kb fragment encompassing the major Bmp4 promoter has been tested in transgenic mice [16], and its activity compared with the expression of the Bmp4-lacZ-neo knock-in reporter mouse [17]. Although this fragment regulated expression in a similar manner to endogenous Bmp4, it failed to drive expression in many sites that normally expressed Bmp4 [16]. In the current study, we therefore generated a reporter construct (p7kb-Bmp4Luc) to monitor Bmp4 expression (Fig. 1A). Luciferase expression controlled by the reporter construct was assessed in NIT-1 (mouse pancreatic β-cell line), αTC1clone6 (mouse pancreatic α-cell line), AR42J (rat pancreatic acinar-cell line) and NIH3T3 (mouse fibroblast cell line) cells transiently transfected with the constructs. Endogenous Bmp4 protein expression levels in these cell lines were determined by western blotting analysis. Bmp4 protein expression in NIT-1and αTC1clone6 cells were significantly higher than in AR42J or NIH3T3 cells (Fig. 1B). Likewise, The level of luciferase expression derived from the transgene was higher in pancreatic NIT-1and αTC1clone6 cells than in AR42J or NIH3T3 cells (Fig. 1C). These results strongly suggest that the region of p7kb-Bmp4Luc suffice to monitor of Bmp4 expression in pancreas.

### p7kb-Bmp4Luc transgenic mice

**Figure 2:**
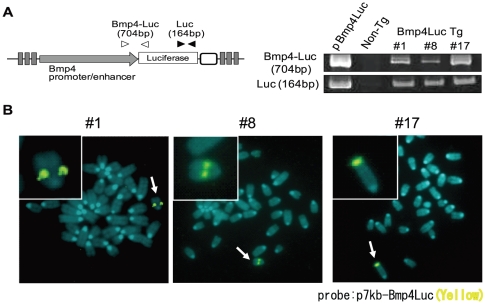
Establishment of 7 kb-Bmp4Luc transgenic mice. (A) Genomic PCR of transgenic mouse lines (#1, #8 and #17) produced by pro-nuclear microinjection of linearized p7kb-Bmp4Luc. Arrowheads indicate primer sets in the left panel. Template DNA was prepared from tails of each transgenic line and from wild-type mice (Non-Tg) and plasmids (pBmp4-Luc). (B) Fluorescence in situ hybridization (FISH) assays were performed using fibroblast cells isolated from the tail of each transgenic mouse line. p7kb-Bmp4Luc plasmid DNA was used as a probe (yellow signals with arrows). Chromosomal DNA was counterstained with 4′,6-diamidino-2-phenylindole (DAPI) (blue).

Luciferase expression from p7kb-Bmp4Luc was selectively observed in pancreatic cells. We therefore generated three transgenic mouse lines (#1, #8 and #17) by pro-nuclear microinjection of linearized p7kb-Bmp4Luc. Insertion of the transgene into these mice was confirmed by genomic PCR (Fig. 2A) and fluorescence in situ hybridization (FISH) analysis (Fig. 2B). The transgene in line #1 and #8 was inserted into chromosome 14, while the transgene in line #17 was inserted into chromosome 1. Genomic PCR showed that insertion of the entire 7-kb promoter region was unreliable, but the presence of the 3-kb region upstream of the translation initiation sites was confirmed by southern hybridization in line #17 (data not shown). Moreover, these results suggest that the biological influence of transgene integration into the host chromosome was small, because strong bioluminescent signals were detected in the pancreas independently in the three transgenic lines, which appeared to be otherwise normal and healthy.

### Bioluminescent signal in pancreas of p7kb-Bmp4Luc-transgenic mice

**Figure 3:**
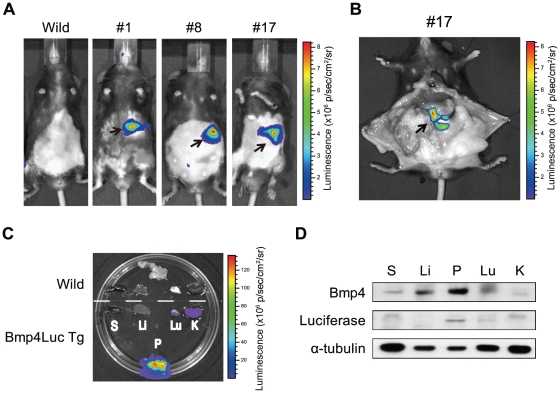
In vivo bioluminescent signals in p7 kb-Bmp4Luc transgenic mice. (A) In vivo imaging assays of the three lines of transgenic mice (#1, #8 and #17). Transgenic mice were anesthetized with 2% isoflurane gas and intraperitoneally injected with D-luciferin. Bioluminescent images were obtained by 1 min exposure in the imaging system. The minimum and maximum photons/second values for each figure are indicated in each rainbow-colored bar scale. Strong bioluminescent signals were detected in the upper abdomen (black arrows) in all three lines in vivo. (B) The strong signal in the upper abdomen was confirmed to be localized to the pancreas (black arrow), by abdominal exploration in a transgenic mouse injected with D-luciferin. (C) Ex vivo imaging assays. Dissected tissues, spleen (S), liver (Li), pancreas (P), lung (Lu) and kidney (K) from wild-type (upper part of the dish) and transgenic mice (bottom part of the dish) were immersed in sterile PBS containing 0.3 mg/ml D-luciferin, and subjected to imaging analysis. (D) Western blot analysis of various tissues from transgenic mice, using antibodies to murine Bmp4, luciferase and α-tubulin.

Luciferase expression in the pancreas in these transgenic mice was monitored by measuring the intensity of the bioluminescent signal (photons/second/region of interest (ROI)), using the Xenogen IVIS Imaging System. There was no detectable bioluminescence in the absence of the luciferase substrate, D-luciferin. However, after intraperitoneal injection of D-luciferin, the bioluminescent signal was detected in vivo in the upper abdomen in all three transgenic mouse lines (Fig. 3A). In contrast, no signal was observed in non-transgenic mice (wild-type) (Fig. 3A). The strong signal in the upper abdomen was confirmed to be derived from the pancreas, by dissection (Fig. 3B). To identify which tissues produced bioluminescent signals, these signals were detected in dissected transgenic mouse tissues incubated with fresh medium and D-luciferin substrate. Bioluminescent signals were detected in the pancreas, lungs, and kidneys, but not in the liver or spleen (Fig. 3C). To determine if the expression pattern of the luciferase proteins was consistent with that of Bmp4, endogenous Bmp4 and transduced luciferase protein expression were examined in various tissues from transgenic mice, using western blotting analysis (Fig. 3D). Bmp4 was abundantly expressed in the pancreas and other tissues, as previously reported, while luciferase expression was notably higher in the pancreas, compared with other tissues. The reason why luciferase expression from the Bmp4 promoter was predominant in the pancreas is unknown. Nevertheless, these results suggested that it was possible to monitor pancreatic-specific Bmp4 expression in these transgenic mice. Strong bioluminescent signals were also detected in the pancreas in lines #1 and #8 (data not shown). All subsequent results refer to line #17 transgenic mice, because this line could be bred more efficiently than the others.

### Expression of Bmp4 and luciferase protein in pancreatic islets

**Figure 4:**
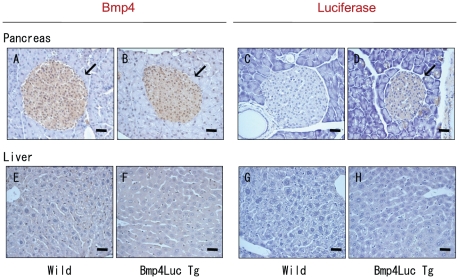
Bmp4 and luciferase proteins in pancreatic islets. Immunohistochemical analysis of pancreas (A–D) and liver (E–H) of wild-type (A, C, E, G) and transgenic mice (B, D, F, H) was performed using antibodies to Bmp4 (A, B, E, F) and luciferase (C, D, G, H). Bmp4-positive cells and luciferase-positive cells are shown as brown cells (arrows) in pancreatic islets. Scale bar shows 25 μm.

We used immunohistochemistry to determine which cells in the pancreas expressed luciferase proteins. Bmp4 is known to be expressed in the adult pancreas, as well as that of the embryo [8]–[10], [18], [19]. Bmp4 protein was expressed in the adult pancreatic islets, and similarly luciferase protein was expressed in islets (Fig. 4A–D). Levels of Bmp4 and luciferase proteins were very low in hepatic cells (Fig. 4 E-H).

### Increase of bioluminescent signal in pancreas after 24-h fasting

**Figure 5:**
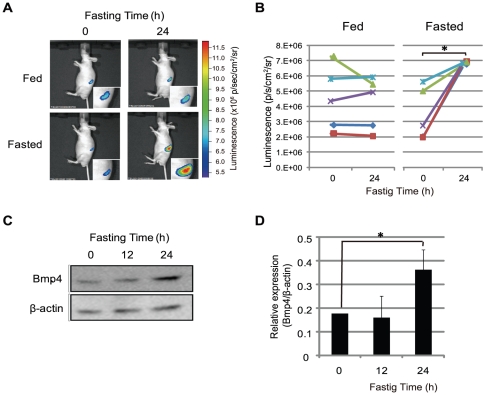
Effect of 24-h fasting on bioluminescent signals in pancreas of p7kb-Bmp4Luc transgenic mice. (A) Transgenic mice subjected to 24-h fasting were injected in the tail vein with D-luciferin (150 mg/kg). At 1-min post-injection, bioluminescent images were obtained by 1-min exposure in the imaging system. The insets show high-magnification views of the pancreas. (B) The bioluminescent signals were quantified using Living Image software, and the intensity of luminescence is shown as photons/second/cm^2^/steradian (p/s/cm^2^/sr), indicated by individual color bars. The data are shown as mean ± SE from three independent experiments. Asterisk (*) represents significant difference (p<0.05) by Student's *t*-test. (C) Western blot analysis of the pancreas from fasted transgenic mice was performed using antibodies to murine Bmp4 and β-actin. (D) Quantification of Bmp4 protein levels by western blotting (C) using an image analyzer. The Bmp4 protein levels were normalized to β-actin as the internal standard.

The pancreas stringently maintains blood sugar concentrations during feeding and fasting by homeostatic regulation. To test if the transgenic mice were able to respond to feeding or fasting, they were fasted for 24 h, followed by determination of bioluminescence (Fig. 5A). The intensity of the bioluminescent signal in the pancreas of fasted mice was increased by about 3-fold, compared with that in fed mice (Fig. 5B). Furthermore, Bmp4 protein levels in the pancreas of mice fasted for 24 h were also increased about 3-fold, compared with those in fed mice, as determined by western blot analysis, normalized to β-actin levels (Fig. 5C,D). These results suggest that Bmp4 expression in the pancreas responds to feeding or fasting, and that it is possible to monitor this pancreatic response in transgenic mice without the need to sacrifice them.

**Figure 6:**
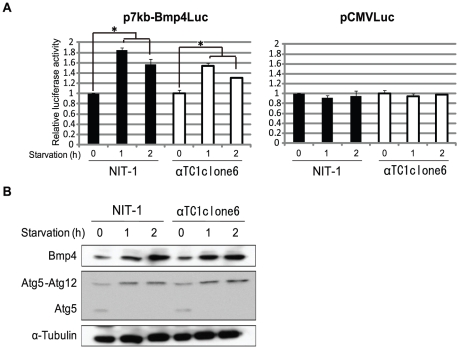
Bmp4 expression in starved pancreatic cell culture in vitro. (A) Luciferase assays of pancreatic cell cultures starved of nutrients. NIT1 and αTC1clone6 cells were transfected with reporter vectors (p7kb-Bmp4Luc and pCMV-Luc), using pRL-TK as an internal control. At 48 h post-transfection, the culture medium was replaced with starvation media (PBS) for 1–2 h, followed by luciferase assay. Relative luciferase activity was normalized to activity from pRL-TK, and showed in ratio based on the activity generated by non-treated cells. The data are shown as mean ± SE from three independent experiments. Asterisk (*) represents significant difference (p<0.05) by Student's *t*-test. (B) Western blot analysis of starved NIT1 and αTC1clone6 cells was performed (A) using antibodies to Bmp4 and the autophagy marker, Atg5-Atg12.

Increased Bmp4 expression in fasted mice was confirmed in pancreatic cell cultures. When NIT-1 and αTC1clone6 cells transfected with the p7kb-Bmp4Luc plasmid were cultured in phosphate buffed saline (PBS) as starvation medium for 1–2 h, luciferase activity driven by the Bmp4 promoter was increased 2-fold in NIT-1 and αTC1clone6 cells (Fig. 6A). In contrast, luciferase expression driven by the cytomegalovirus promoter (pCMV-Luc) did not respond to nutrient starvation in either NIT-1 or αTC1clone6 cells (Fig. 6A). Increased Bmp4 expression was confirmed by western blot analysis. Interestingly, Bmp4 expression was increased in starved NIT-1 and αTC1clone6 cells (Fig. 6B). These results indicate that the factor/s involved in fasting is/are associated with the regulation of Bmp4 expression in pancreatic β and α cells.

Starvation-induced autophagosomes engulf the cytosol and/or organelles and deliver them to lysosomes for degradation, thereby resupplying depleted nutrients [20], [21]. To further assess the induction of autophagy under poor nutritional conditions, the expression of the autophagy-related Atg5-Atg12 complex was detected by western blotting. Atg5 protein levels decreased, while the Atg5-Atg12 complex increased under poor nutritional conditions (Fig. 6C). These results confirm that starvation induces autophgy and BMP4 expression simultaneously, and suggested that Bmp4 plays some roles in the autophagy process.

## Discussion

Mammalian Bmp4 is located within a large uncharacterized region of the genome over 1 Mb in length, known as a “gene desert” [22]. It is highly conserved between some mammalian species, but little is known about the transcriptional regulation of the Bmp4 gene. The Bmp4 gene has at least two major transcription start sites, resulting in the production of slightly different mRNAs [23]. A construct containing 2.4 kb upstream from the first transcription start site has been tested in transgenic mice as the major Bmp4 promoter (the Bmp4-lacZ-neo knock-in reporter mouse) [16], [17]. Although this fragment regulated expression in a similar way to endogenous Bmp4 in tooth ameloblasts and developing hair follicle shafts and matrix, it failed to drive expression in many sites that normally express Bmp4, such as bone, nasal cartilage, limb buds, ear, and brain [16]. The inability of this 2.4-kb Bmp4 construct to recapitulate the complete in vivo expression pattern of the gene indicates that important cis-acting regulatory elements must be located outside of this region [16]. A 46-kb upstream region of the mouse sequence containing evolutionarily-conserved regions has been confirmed as a mesoderm-specific enhancer element in a previous study of transgenic mice [24]. To evaluate the gene expression of Bmp4, we constructed two novel reporter vectors utilizing the 7-kb enhancer/promoter region, together with 5′-noncoding exons and introns of the Bmp4 gene. It is noteworthy that this reporter is strongly expressed in pancreas islet cells and regulated by nutrient circumstance.

Bmp4 expression in the pancreatic islets has been reported to occur especially in β cells [8], [9]. β cells constitute 90% of the islets, and the localization of luciferase expression in this study was therefore consistent with previous results. Bmp4 and Bmp Receptor 1A (BmpR1A) are expressed in pancreatic β cells, and autocrine Bmp4 through Smad signaling is involved in insulin gene expression, proinsulin processing, glucose sensing, secretion-stimulus coupling, incretin signaling, and insulin exocytosis [8], [9]. On the other hand, a recent report showed that Bmp4 stimulation blocked the differentiation and promoted the expansion of endocrine progenitor cells, thereby revealing a novel paradigm of signaling explaining the balance between expansion and differentiation of pancreatic duct epithelial progenitors [10].

Autophagy induced by deprivation of nutrients is an evolutionarily-conserved lysosomal degradation pathway in which the cell self-digests its own proteins and organelles, and thus maintains macromolecular synthesis and ATP production [21]. β-cell-specific autophagy-deficient mice show hypoinsulinemia and hyperglycemia [25], [26]. Autophagy is required to maintain the structure, mass and function of pancreatic β cells [27], [28]; accordingly, expression of LC3 (autophagy marker)-positive cells was increased in the pancreas of 24-h-fasted transgenic mice. Our observations suggest that fasting or starvation stress in pancreatic cells independently induces both an autophagy response and Bmp4 expression, or that autophagy signals induce Bmp4 expression. Further studies are needed to clarify this issue.

Type 2 diabetes is characterized by abnormal regulation of nutrients and their metabolites, and develops as a consequence of combined insulin resistance and relative insulin deficiency [29]. Insulin and its downstream signal molecules, such as mTOR, are well-known inhibitors of autophagy [30], whereas glucagon, a counter-regulatory hormone of insulin, induces autophagy [31]. Type 2 diabetes models based on p7kb-Bmp4Luc transgenic mice may be suitable for evaluating disease onset, and these transgenic mice may provide a useful tool for future toxicological screening studies, and for further studies to reveal the functions of Bmp4 in the pancreas.

## Materials and Methods

### Ethics Statement

All of the animal experiments described were approved by the Institutional Animal Care and Use Committee, Tottori University (permission number: 21-2-48 and 09-Y-70). All mice in this study were received humane care in compliance with Tottori University's guidelines for the care and use of laboratory animals in research, fed ad libitum and housed in a room maintained at a constant temperature of 22°C, at 50% humidity, and with a 12-h light-dark cycle.

### Construction of reporter plasmids to monitor expression of Bmp4

The transgenes were assembled using the genomic DNA 7-kb upstream region from the transcription start site of the mouse Bmp4 gene (GeneID: 12159), which contains the promoter and enhancer, as well as 5′ noncoding exons and introns. The translation start site of firefly luciferase (pGL3-Basic; Promega, CA, USA) was adjusted to that of Bmp4. The Bmp4Luc expression unit was flanked on either side with insulators derived from the chicken β-globin gene. pCMV-Luc (cytomegalovirus early gene promoter) and p0Luc (without promoter) were constructed as control vectors.

### Cell culture and treatment

The NIT-1 (CRL-2055: ATCC, MD., USA), mouse β cell line was grown in monolayers in Ham's F-12K (Kaighn's Modification) medium (Wako Pure Chemical Industries, Osaka, Japan) supplemented with 10% heat-inactivated fetal calf serum (FCS). The αTC1clone6, (CRL-2934: ATCC), mouse α cell line was grown in Dulbecco's modified Eagle's Medium (DMEM) (Nissui Seiyaku, Tokyo, Japan) containing 10% heat-inactivated FCS plus 15 mM HEPES (Wako Pure Chemical Industries), 0.1 mM non-essential amino acids (Wako Pure Chemical Industries), 0.02% bovine serum albumin (Wako Pure Chemical Industries), and 2 g/l glucose (Wako Pure Chemical Industries). The AR42J (CRL-1492: ATCC) acinar cell line was grown in monolayers in Ham's F-12K medium supplemented with 20% FCS. NIH3T3 (RCB0150; RIKEN Cell Bank, Wako, Japan) mouse fibroblast cells were grown in DMEM containing 10% FCS. Cultures were maintained with 0.2% penicillin-streptomycin (Invitrogen, Carlsbad, CA, USA) at 37°C in a water-jacketed incubator with 5% CO_2_, and subcultured at a ratio of 1∶3 with 0.1% trypsin-EDTA every 3–4 days, when cells reached about 80% confluence. Cells at passages 3–10 were used in this study. Starvation of culture cells was induced by incubation in PBS for 1–2 h.

### Measurement of luciferase activity

Luciferase activity in vitro was determined using the luciferase reporter assay system (PicaGene; Toyo Ink, Tokyo, Japan). Cells were plated into 6-well dishes and co-transfected with p7kb-BmpLuc plus pRL-TK (PicaGene; Toyo Ink) control vector using lipofectamine LTX reagent (Invitrogen), according to the manufacturer's instructions. After removal of lipofectamine from the medium 48 h later, luciferase activity was quickly measured using a lumicounter ATP-300 (Advantec, Tokyo Japan) or AB-2550 Kronos Dio (Rose Scientific Ltd, Alberta, Canada) for 10 seconds, repeated three times.

### Generation of p7kb-Bmp4Luc transgenic mice

Three lines (#1, #8 and #17) of transgenic mice were generated by pronuclear microinjection of linearized p7kb-Bmp4Luc DNA into BDF1×BDF1 fertilized eggs. The mice used in the studies described here were heterozygous for the transgene, and age-matched (8–15-week-old) male mice. All mice were bred and housed under a 12 h light-dark cycle with free access to food and water.

### Genomic PCR and FISH

The transgenes in the mice were examined by PCR using tail DNA, and by FISH using fibroblast cells isolated from tails of p7kb-Bmp4Luc transgenic mice. The Bmp4 promoter- and luciferase-specific PCR primers used were 5′-GTTTGTTCAAGATTGGCTCCC-3′ and 5′-CGTTTCATAGCTTCTGCCAAC-3′. The PCR product was 1.2 kb, and the PCR conditions were: initial denaturation at 94°C for 2 min, and 35 cycles of denaturation at 94°C for 1 min, annealing at 60°C for 1 min and extension at 72°C for 1.5 min, and a final extension at 72°C for 7 min using Taq DNA polymerase (BioAcademia). Other PCR primers were luciferase-specific primers 5′-ATCCATCTTGCTCCAACACC-3′ and 5′-TCGCGGTTGTTACTTGACTG -3′. The PCR product was 164 bp and the PCR conditions were: initial denaturation at 94°C for 2 min, and 35 cycles of denaturation at 94°C for 30 sec, annealing at 55°C for 30 sec and extension at 72°C for 24 sec, and a final extension at 72°C for 7 min using Taq DNA polymerase. FISH was carried out according to standard protocols with a fluorescein isothiocyanate-labeled p7kb-Bmp4Luc probe.

### Bioluminescent imaging

Bioluminescent optical imaging was performed using a Xenogen IVIS 200 imaging system (Xenogen, Alameda, CA, USA). Transgenic mice were anesthetized with 2% isoflurane gas and 15 mg/ml D-luciferin (Promega) in sterile PBS (150 mg/kg body weight) was injected into the tail vein. Bioluminescent images were obtained at 1 min post-injection by 1 min exposure in the imaging system. The minimum and maximum photons/second for each figure are indicated in each rainbow-colored bar scale.

### Western blotting

Frozen mouse tissues were disrupted using a Multi**-**Beads Shocker (Yasuikikai, Osaka, Japan), and homogenized on ice in extraction buffer (10 mM Tris-HCl (pH 7.6), 15 mM KCl, 1 mM EDTA, 0.5 mM phenylmethylsulfonyl fluoride) and centrifuged for 30 min at 4°C. The protein concentration was determined using a Bio-Rad protein assay kit (Bio-Rad, Hercules CA, USA). Fifteen micrograms of protein were separated by 10% sodium dodecyl sulfate-polyacrylamide gel electrophoresis and then transferred to a polyvinylidene fluoride membrane (Millipore, MA, USA). The membrane was blocked with 2% skim milk for 30 min and then incubated overnight with rabbit polyclonal anti-Bmp4 antibody (1∶1,000 dilution, Abcam, Cambridge, MA, USA), goat polyclonal anti-luciferase antibody (1∶1,000 dilution, Promega), rabbit polyclonal anti-Atg5 antibody (1∶500 dilution, Sigma, St. Louis, MO, USA), rabbit polyclonal anti-LC3 antibody (1∶500 dilution, Sigma), mouse monoclonal anti-α tubulin antibody (1∶2,000 dilution, Sigma) and mouse monoclonal anti-β-actin antibody (1∶4,000 dilution, Abcam). The membrane was incubated with rabbit anti-mouse and anti-goat anti-rabbit IgG-horseradish peroxidase (1∶ 2,000 dilution, Santa Cruz Biotechnology, Santa Cruz, CA,USA) for 2 h and developed by enhanced chemiluminescence using the ECL Detection Kit (GE Healthcare, Piscataway, NJ, USA) with an image analyzer (LAS-1000 plus; Fuji Photo Film Co. Tokyo Japan).

### Immunohistochemistry

Tissues were fixed in 10% formaldehyde overnight at room temperature and embedded in paraffin, and 10-μm sections were applied to slides. The primary antibodies used were rabbit polyclonal anti-Bmp4 (1∶200, Abcam), goat polyclonal anti-firefly luciferase (1∶50, Promega), rabbit polyclonal anti-LC3 (1∶200, Sigma). An avidin-biotin immunoperoxidase system (Nichirei, Tokyo, Japan) was used to visualize antibodies bound to the tissue. Samples were counterstained with hematoxylin.
